# Hepatitis B functional cure and immune response

**DOI:** 10.3389/fimmu.2022.1075916

**Published:** 2022-11-17

**Authors:** Jia-Rui Zheng, Zi-Long Wang, Bo Feng

**Affiliations:** Beijing Key Laboratory of Hepatitis C and Immunotherapy for Liver Diseases, Peking University People’s Hospital, Peking University Hepatology Institute, Beijing, China

**Keywords:** chronic hepatitis B, functional cure, innate immunity, adaptive immunity, antiviral

## Abstract

Hepatitis B virus (HBV) is a hepatotropic virus, which damage to hepatocytes is not direct, but through the immune system. HBV specific CD4^+^ T cells can induce HBV specific B cells and CD8^+^ T cells. HBV specific B cells produce antibodies to control HBV infection, while HBV specific CD8^+^ T cells destroy infected hepatocytes. One of the reasons for the chronicity of HBV infection is that it cannot effectively activate adoptive immunity and the function of virus specific immune cells is exhausted. Among them, virus antigens (including HBV surface antigen, e antigen, core antigen, etc.) can inhibit the function of immune cells and induce immune tolerance. Long term nucleos(t)ide analogues (NAs) treatment and inactive HBsAg carriers with low HBsAg level may “wake up” immune cells with abnormal function due to the decrease of viral antigen level in blood and liver, and the specific immune function of HBV will recover to a certain extent, thus becoming the “dominant population” for functional cure. In turn, the functional cure will further promote the recovery of HBV specific immune function, which is also the theoretical basis for complete cure of hepatitis B. In the future, the complete cure of chronic HBV infection must be the combination of three drugs: inhibiting virus replication, reducing surface antigen levels and specific immune regulation, among which specific immunotherapy is indispensable. Here we review the relationship, mechanism and clinical significance between the cure of hepatitis B and immune system.

## Introduction

The infection of chronic hepatitis B virus (HBV) is still a worldwide problem that seriously threatens human health. It is the principal reason of end-stage liver diseases, including cirrhosis and hepatocellular carcinoma (HCC). As WHO estimated, there were 290 million people infected with chronic HBV all over the world in 2019, and about 1.5 million people were newly infected each year (1). The prevalence rate of HBsAg among the general people in China is 6.1%, and there are about 86 million people with positive HBsAg (2). More than 90% of HCC in China is related to chronic HBV infection.

At present, there are two kinds of widely used anti-HBV drugs, namely nucleos(t)ide analogues (NAs) as well as peginterferon-α (PegIFNα). The treatment goal determined by domestic and foreign guidelines is to improve the long-term outcomes by maximizing the persistent inhibition of HBV (3–5). The goal of CHB treatment is to pursue cure. According to HBV related biomarkers and possible outcomes, hepatitis B cure can be divided into partial cure, functional cure (clinical cure) and complete cure (6). Because of covalently closed circularDNA (cccDNA) existence and integrated HBV DNA in hepatocytes, HBV cannot be completely eliminated, but functional cure, that is, the clearance of HBsAg, becomes possible. The so-called functional cure refers to achieving HBsAg seroclearance, with or without positive anti-HBs, based on continuous undetectable HBV DNA and HBeAg after a finite course of treatment. It can be originated from the induction of PegIFN based regimens or NAs, and the spontaneous clearance of HBsAg (7).

Chronic hepatitis B (CHB) is an immune related disease. HBV does not directly damage the liver but through abnormal immune response. The interaction between the replication of virus and the antiviral immunity of host determines the final result of HBV infection. The responses of host’s innate and specific immunity to the virus promote the clearance of HBV in patients with acute hepatitis B. HBV continues to replicate and the host immune response is insufficient in patients with CHB. Integration of HBV DNA in hepatocytes, persistent presence of HBV cccDNA, dysfunction of T cells and insufficient response of B cells are the major obstacles to eliminate HBV. This determines that CHB cannot be completely cured through direct antiviral agents as chronic hepatitis C. Therefore, in order to cure hepatitis B, it is more important to restore host immune function in addition to inhibiting virus replication, reducing or even eliminating virus antigens (8). Here, we focuse on the chronicity of HBV infection, the relationship between CHB functional cure and host innate immunity, specific cellular and humoral immunity, and the possible role of new immunomodulators in functional cure.

## Persistent HBV infection and host immune response

### Persistent HBV infection and host innate immune response

As a crucial immune organ of the human body, the liver is rich in natural killer cells (NK cells), natural killer T cell (NKT cells), as well as macrophages (eg. Kupffer cells), of which NK cells and NKT cells making up half in terms of numbers of lymphocytes in the liver. As the first line of defense against HBV, the natural immune system is greatly important in the initial and chronic process of HBV infection. In the process of early virus clearance and specific immune response, the innate immune response is greatly important, whose deficiency is an important reason for presistent HBV infection. HBV has evolved an active strategy to actively evade the natural immune recognition or actively interfere with the natural immune signaling pathway to induce immune suppression, which is conducive to self-replication (9).

Age at exposure contributed greatly to the chronicity of the human HBV infection, which is associated with the immature development of the innate immunity in perinatal and infant periods and the unstable intestinal microbiota (10). Lebossé et al. conducted a study involved 105 untreated CHB patients to explore the expression profiles of 67 genes, which were involved in the innate immune pathways in the hepatic tissues of 105 untreated CHB patients. The results proved that the expression profile of genes in the liver of CHB patients showed significant down-regulation of antiviral effectors, IFN stimulating genes (ISGs), Toll-like and pathogen recognition receptors (PPP) pathway genes compared with that of the healthy control group. In CHB patients with negative HBeAg, some genes expression was negatively correlated with the level of qHBsAg, but not directly related to HBV replication. It is suggested that the innate immunity in the liver of CHB patients is seriously impaired, especially at high levels of HBsAg (11). More and more studies have found that viral antigens, including HBsAg, is crucial to suppress natural immunity and promoting viral immune escape. HBsAg can inhibit stimulator of interferon genes (STING) expression in NK cells and inhibit NK’s response to cGAMP (12). Interaction of HBsAg as well as TGF β- Activated kinase 1 (TAK1) - TAB2 complex s inhibits NF-κB signal pathway by reducing post-translational modification and autophagic degradation (13). HBsAg promotes the production of GP73 and HBV replication by inhibiting NF-κB signaling pathway (14). HBsAg inhibits interferon regulatory factor (IRF)-α and Toll-like receptor 9 (TLR9) nuclear translocation, thereby specifically inhibiting TLR9 mediated interferon production. HBsAg can directly cause dysfunction of bone marrow dendritic cells in CHB patients, which is regarded as a potential mechanism for HBV escaping from the immune system (15). These outcomes reveal new insights into the mechanism of the persistence of HBV in the liver. Methods aimed at reducing the level of HBsAg may restore the immune response upon the virus, which are considered as one of the strategies to cure CHB.

### Persistent HBV infection and cellular immunity

In the liver puncture samples of CHB patients, inflammatory necrosis and fibrosis of different degrees were found in and around the portal area of the liver and the infiltrating inflammatory cells were mainly lymphocytes, indicating the great importance of immune cells in its pathogenesis (5). T cell exhaustion (Tex) is one of the characteristics of CHB patients. It means that when the virus is chronically infected, owing to being exposed to persistent antigens and an inflammatory environment for a long time, T cells have a constantly stimulation and gradually lose their effective function, and memory T cell characteristics begin to be lost. Tex cells have independent differentiation pathways, phenotypes, functions, and subpopulation dynamics. In addition to the decrease in number, its functions such as rapid growth and cytokine secretion are lost in a gradual and graded manner (16). Currently, it is recognized that it may be the enhanced expression of inhibitory molecules, such as programmed cell death protein-1 (PD-1) and cytotoxic T lymphocyte-associated antigen-4 (CTLA-4) (17). Studies have found that by blocking the interaction between PD-1 and programmed death ligand-1 (PD-L1), Tex can be partially reversed (18). In addition, the transcriptional programs and metabolic patterns of Tex cells have also changed. Based on the latest results of single-cell transcriptome analysis, study has found that the exhausted CD8^+^T cells of hepatitis B patients at different stages come from different sources, in which patients at the immune activation stage mainly come from autologous clonal expansion, while patients at the recovery stage of acute infection are more from the transformation and infiltration of peripheral effector T cells (19).

CD8^+^T cells play an important role in cellular immunity, which are considered to be effectors of virus clearance, killing HBV-infected hepatocytes with antigenic specificity restricted by MHC-I molecules (20). A few studies have revealed that in patients with acute hepatitis, activated CD8^+^T cells targeting HBsAg are abundant, but in patients with chronic hepatitis B, the number of CD8^+^T cells is reduced and their functions are impaired, leading to persistent viral replication (21, 22). In the chimpanzee model infected with HBV, a considerable delay in viral clearance was observed by using CD8^+^T cells blocking antibodies (23). The immune response of HBV-specific CD8^+^T cell relies on the development of the disease course and is greatly susceptible to the influence of the level of HBV replication. For patients with viral load>107 copies/ml, it is difficult to detect CD8^+^T cells, whose deletion mechanism is still unclear, but it has been reported that it may be related in part to increased apoptosis of CD8^+^T cells (24). A recent research has explored the causes and found that HBV can eliminate immature HBsAg-specific T cells in the thymus through monocytic myeloid derived suppressor cells (mMDSCs), thus forming an HBV-specific T-cell tolerance environment (25).

CD4^+^T cells have significant heterogeneity, in terms of their cytokine expression, CD4^+^T helper cells (Th) can be classified into Th1, Th2, Th17, and regulatory T cells (Treg) subsets (26). Maintaining a balance in these T cells is crucial to liver immune homeostasis. The depletion of HBV-specific CD4^+^T cells and the imbalance of regulatory T cells may be significant features of CHB (27). Although the pathogenesis of T cells in different stages of HBV is still poorly understood at present, the rise of single-cell sequencing technology may provide new insights for delineating the profile of liver immune cells in different stages of HBV and exploring the mechanism (19).

Some studies have shown that the expression of Treg and Th17 cells greatly increased in CHB patients (28, 29). Compared with convalescent patients and healthy people, the proportion of peripheral blood Treg cells had a significant increase in CHB patients, and the proportion as well as the number of Treg cells positively correlated with serum HBV DNA content (30). Treg cells contributed a lot to maintain the immune tolerance and inhibit the activation and proliferation of T lymphocytes and the maturation of dendritic cells (DC) (31). In addition, Treg could also inhibit the ability of T follicular helper cells (Tfh) through its expression of CTLA-4, resulting in its inability to assist the functional maturation of B cells to produce neutralizing antibodies, leading to persistent HBV infection (32).

### Persistent HBV infection and humoral immune response

Over the past 20 years, most researches on the pathogenesis of HBV have paid attention to the T cell responses and the chronic phase clearance deficiency caused by virus specific T cell failure, while B cells have been neglected for a long time (33). Actually, humoral immune responses mediated by B cells are important for controlling and clearing HBV, whose infection has a great influence on the secretion of HBV-specific antibody and compartment of global B cells (34). The reactivation of HBV most often occurs in patients receiving cancer chemotherapy, especially those receiving B cell depleting agents, for example, rituximab and orfatumumab for blood or solid organ malignancies and patients receiving hematopoietic stem cell transplantation without antiviral prevention (35). One major way in which B cells are involved in anti-HBV infection is the production of specific antibodies against diverse HBV protein components, such as antibody to hepatitis B s antigen (HBsAb), antibody to hepatitis B e antigen (HBeAb) and antibody to hepatitis B core antigen (HBcAb). HBcAb and HBeAb are considered as the marekers for the diagnosis of HBV infection, while only HBsAb can recognize and bind to HBsAg (36), thus playing an essential part in the clearance of HBsAg (37). HBsAb can not only prevent HBV from entering the body through acting as a protective neutralizing antibody to combine with free HBV virus particles, thus reducing the viral load *in vivo* (38), but also eliminate infected cells by mediating antigen dependent cytotoxicity and phagocytosis (39). In chronic HBV infection, global peripheral B cells are activated and hypofunctioning, while B cells which secrete anti-HBs are rarely detected (40). A recent study enrolled 13 healthy individuals without vaccination (HBsAg, HBsAb and HBcAb are negative) and 38 treatment naïve CHB patients using RNA sequencing of B cells showed that, compared with peripheral B cells, a upregulation of B cell receptor pathway was seen in liver B cells (41). Zhou et al. found that in CHB patients, the expression of skewed CD39 and CD73 on B cells was correlated with a high viral burden, liver inflammation and antiviral efficacy, and the skewed CD39/CD73/adenosine pathway played an essential part in the hyperactivation of B cells (42). Additionally, although in CHB patients could detect a significantly higher total immunoglobulin G (IgG) in the serum than that in healthy individuals, HBV-specific antibodies were lacking (34, 43). The immune dysfunction in CHB patients might be related to B cells hyperactivation, impaired differentiation, inhibitory signal as well as regulatory B cells activation (44). A recent study isolating CD19^+^B cells from peripheral blood of 4 healthy controls and 4 CHB patients, and from the B cell transcriptome of all participants, totally 1401 genes with different expression were identified. The results revealed that B cells function of CHB patients was impaired, with the increasing expression of TLR4, the activation of NF-κB pathway, as well as the alteration of mitochondrial function (45). During the persistent infection of HBV, the enrichment of IL-35^+^B cell could be seen in CD19^+^CD24^hi^CD38^hi^ B cell. Meanwhile, Breg cells caused dysfunction of T cells through IL-35 dependent mechanism, as well as the intercellular contact exerted dysregulation (46).

Additionally, studies suggested that Tfh, which regulates B cells-mediated humoral immune response, is phenotypically different, and leads to deficiency in humoral immunity of CHB patients (39). Tfh cells are located in secondary lymphoid organs and promote the differentiation of B cells by secreting IL-21 (47). The reaction of Tfh cells to HBsAg to produce IL-21 is defective during CHB. However, despite of the low levels of IL-21, Tfh cells can effectively do a favor to B cell responses by producing IL-27 despite low levels of IL-21, which directs naive and memory B cells to form plasmablasts as well as plasma cells by enhancing B lymphocyte-induced maturation protein-1 (48). Ayithan et al. found that treatment of cytokine IL-21 induced by TLR8 agonists on peripheral blood mononuclear cells (PBMCs) in CHB patients was achieved by enhancing Tfh cells co -expressing IL-21^+^BCL-6^+^ and ICOS+BCL-6^+^ (49). Tfh cells contribute to differentiate B cells into antibody producing plasma cells and provide lifelong protection (50). Evidence have proved that depletion of TFh cells is a significant cytokine for B cells maturation and clearance of chronic viral infections (16, 51).

## Anti-viral therapy and host immune response

### Anti-viral therapy and host innate immune response

Conventional IFN α has been used to treat CHB for more than 30 years, and was replaced by PegIFNα in 2005. It has anti-HBV and immunomodulatory effects, and plays a variety of key roles in innate and adaptive immune responses (52). Many studies have proved that the therapeutic effect of PegIFNα for its induction of the repression of specific interferon-stimulated genes (ISGs) and secretion of antiviral proteins (53, 54).

IFNα can drive the proliferation, activation and antiviral ability of NK cells *in vivo*, mainly showing that the activated CD56^bright^ NK cells significantly expand, thereby indirectly inhibiting HBV replication, but this effect will be reduced after the treatment cessation (55). In HBeAg positive CHB patients who experienced 48 weeks of PegIFNα treatment, CD56^bright^ NK cells of sequenced NAs treatment were still higher than the baseline level, while PegIFNα could promote its expansion and increased activating receptors NKp30 and NKp46 expression in NK cells. NK cells activation and proliferation in CHB patients under PegIFNα treatment followed by NAs were significantly stronger than those with NAs or PegIFNα alone (56).

A prospective cohort study analyzed the changes of NK cells at week 12 and 24 in CHB patients received PegIFNα2α and entecavir (ETV) treatment. In patients treated with PegIFNα, a increase was seen in the frequency of CD56^bright^ NK cells, while there was a decrease in that o f CD56^dim^ NK cells, and the expression of NKp46 and IFNAR2 receptors increased. In patients treated with ETV, although the frequency of NK cells increased, no difference was found in that of CD56^bright^ and CD56^dim^ NK cells and the expression of IFNAR2 at baseline and after treatment. Based on the 60% decrease of HBsAg level from baseline, patients were classified into responders and non-responders. In PegIFNα responders, the frequency of CD56^bright^ NK cells and the expression of IFNAR2 increased significantly after treatment compared with that of the baseline, while there were no changes in PegIFNα non-responders and ETV treatment responders. In patients with ETV treatment, NK cell frequency increased significantly, while the frequency of NKp46^bright^ and IFNAR2^+^NK and IFNAR2 MFI decreased greatly at 12 and 24 weeks. It is suggested that in CHB patients, PegIFNα could significantly enhance the frequency and function of NK cells compared with ETV treatment, and the efficacy of IFNα or ETV is associated with the improvement of NK cell function (57). A meta-analysis found that there was no significant difference in NK frequency between CHB patients before and after NAs antiviral therapy, and the activated receptor was up-regulated, while the inhibitory receptor was similar to the healthy control (58).

### Anti-viral therapy and cellular immunity

PegIFNα plays a dual role of immune regulation and antiviral as mentioned above and NAs can strongly interfere with HBV replication as a reverse transcriptase inhibitor, which is easy to use and well tolerated (59). However, it is a pity that the clearance effect of both drugs on HBsAg is not ideal. Therefore, exploring the immune changes of the host after drug treatment and find an immunotherapy scheme may provide new insights into improving the therapeutic effect of patients and achieving clinical cures as much as possible.

Targeted recovery of exhausted T cells may be the focus of HBV treatment in the future, and several studies have been explored accordingly. Among them, blocking agents of immune checkpoints like PD-1/PD-L1 can alleviate the down- regulation of specific T cells and resuscitate the exhaustion of T cells (18). Blockers of some other checkpoint molecules, like T-cell immunoglobulin domain and mucin domain-containing molecule-3 (TIM-3) and CTLA-4, can restore virus-specific CD8^+^ T-cell responses in CHB patients. Inhibitors of metabolic checkpoints may also be an effective means to restore T cells exhaustion. Acyl coenzyme A-cholesterol acyltransferase (ACAT), an enzyme that regulates cellular cholesterol, its inhibitors can improve the ability of antiviral T cells to clear the virus and rescue exhausted T cells (60). In addition, research has proved that the use of T cell immunotherapy can achieve effective control of HBV (61).

The ideal antiviral therapy for CHB patients may eventually lead to the effective recovery of HBV-specific T cells frequency and function (62). Among them, virus-specific CD8^+^T’s number and function recovery might be a research hotspot of current therapy (63). Several studies have shown that the cytotoxic and non-cytotoxic abilities of virus-specific CD8^+^T cells are significantly enhanced with a decreased viral load after antiviral treatment, which is closely associated with HBeAg negative conversion. Some researches have also focused on the exploration of the effect of new therapeutic drugs on CD8^+^T cells (64, 65). These results indicate an interaction between antiviral therapy and virus-specific cellular immune function, and the regulation of immune cells can improve the immune microenvironment and further affect antiviral therapy.

The negative regulation of Treg cells can contribute to the deficiency and absence of HBV-specific T cell response, which is an important mechanism of CHB persistence. Several studies have shown that the proportion of Treg cells had a close association with the clinical efficacy of PegIFNα (66). In the course of antiviral treatment, it has been found that NAs could lead to a decrease in Treg cell levels, which may be related to the targeting effect of NK cells (67). In addition, it can also inhibit the level of virus-specific Th17 cells in the peripheral blood (67), and the serum levels of IL-22 and IL-23 also decreased (68), which may be related to direct viral inhibition and inflammation regulation. The restoration of Th17/Treg balance may also be important for reducing HBsAg levels in patients (69).

An important feature of CHB patients is the decreased number and changed ratio of Th1 and Th2 cells . Several previous research have proved that the number of Th1 and Th2 cells and the expression of related cytokines increased after antiviral treatment (70), which is related to the decrease of HBV DNA viral loads (71). However, compared with Th1 and Th2 cells of chronic HBV patients, Treg cells and Th17 cells are more sensitive subtypes of HBV replication inhibition induced by ETV (70).

### Anti-viral therapy and humoral immune response

Except for the proven effect of PegIFNα on natural killer cells and T cells (55), B cells are greatly important in this process as well (72). PegIFNα treatment may play an immunomodulatory role through remodeling the B cell compartments, which is related to continously increased sCD30 levels and decreased plasma HBsAg (73). HBcAb is an important indicator of host humoral immune response. A retrospective cohort study lasted for 2 years showed that CHB patients whose HBV DNA <9 log10 copies/mL and anti-HBc ≥4.4 log10 IU/mL at baseline level had 37.1% and 65.8% HBeAg seroconversion rates under NAs and PegIFN treatment, respectively (74). Another study involved 139 Chinese patients receiving entecavir or entecavir maleate treatment for 240 weeks also showed that 25.2% of the patients reached the goal of serological response (HBeAg seroconversion), at the same time HBcAb level of these patients increased significantly at 240 weeks (75). A recent research showed that a *de novo* higher level of HBcAb was related to the sustention of response, the decline of HBsAg, the clearance of HBsAg and the loss of HBeAg after *de novo* PegIFNα (p < 0.050), and for patients received add-on PegIFNα, higher HBcAb was related to the loss of HBeAg (p = 0.012) (76). Thus, baseline HBcAb titre can be concerned as an effective predictor of the efficacy of PegIFNα and NAs therapy in CHB patients with positive HBeAg.

## CHB functional cure and host immune response

### CHB functional cure and host innate immune response

PegIFNα combined with NAs treatment significantly affects the f unction and phenotype of NK cells, thereby playing an important role in clearing HBsAg. Stelma et al. followed up CHB patients under PegIFNα treatment combined with adefovir for 48 weeks, and analyzed the function and phenotype of NK cells in 7 patients with functional cure, 7 matched patients without functional cure, and 7 healthy controls. They found that t he absolute number and the proportion of CD56^bright^ NK cells had a significant increase, while those of CD56^dim^NK cells had a decrease in CHB patients received combination therapy. Compared with those without functional cure, t he expression of chemokine receptor CX3CR1 on CD56^bright^ NK cells and the suppressor receptor NKG2A on CD56^dim^NK lymphocytes decreased significantly at baseline, and higher CD56^bright^ TNF-related apoptosis-inducing ligand (TRAIL) expression and IFNγ were obtained at the end of treatment in patients with functional cure, compared with those without it (72). Inactive HBsAg carriers (IHC), which were previously believed to require no treatment, can achieve higher HBsAg seroconversion rate after PegIFNα. Cao et al. compared the changes of NK cells in IHCs with and without the seroconversion of HBsAg after 48 weeks of PegIFNα therapy. They found that the proportion, the IFNγ secretion and CD107a expression of NK cells were greatly higher in HBsAg seroconversion group compared with those without HBsAg seroconversion both at baseline and during PegIFNα treatment. In addition, NK cell activity had an increase in patients with HBsAg seroconversion group, especially before the seroconversion of HBsAg, which were not seen in patients without HBsAg seroconversion. In summary, PegIFNα induced NK cells to increase with enhanced activity, which is conducive to the HBsAg seroconversion of IHCs (77).

NAs play a direct antiviral role mainly by inhibiting HBV DNA polymerase. Does it affect the immune function of the body? Liu et al. conducted immunological studies on CHB patients with decreased HBsAg levels, HBV DNA<1000 IU/mL and negative HBeAg levels after NAs treatment. Compared with untreated CHB patients, the proportion of total NK cells, CD56^dim^NK cells increased, and that of CD56^bright^NK cells decreased in these patients, but there was no significant difference in the healthy controls. It suggested that peripheral blood CD56^dim^ NK cells recovered after inhibition of virus replication, which could be conducive to reduction of the HBsAg levels in CHB patients (69). The potency of TDF antiviral therapy upon the frequency of NK cells in peripheral blood in pregnant women whose HBV DNA was positive was analyzed. The frequency of NK cells, NKp46^dim^ NK cells and CD56^bright^ NK cells after delivery was markedly higher than that before delivery both in the treatment and untreated groups. Compared with untreated group, a significant increase of the frequency of NK cells and CD56^bright^ NK cells after delivery could be seen in the treatment group, while that of CD56^dim^ NK cells was significantly lower. The frequency of postpartum NK cells increased significantly in pregnant women under antiviral treatment. Unfortunately, the impact of TDF on HBsAg levels was not analyzed (78). After NAs treatment, the body immunity can be partially recovered. Then, for CHB patients treated with NAs and low HBsAg levels, whether adding Peg IFN has stronger immune effect than continuing NAs treatment? A recent study proved that for CHB patients with low HBsAg levels who received NAs treatment, the addition of PegIFNα was more likely to lead to the decrease or even elimination of HBsAg by increasing the activity of CD56^bright^NK cells (79).

Several studies have shown that CHB patients who comply with the guideline recommendation to discontinue NAs therapy may have HBsAg seroclearance (80, 81). The best target is CHB patients with no liver cirrhosis, undetectable HBV DNA and HBeAg, as well as low HBsAg levels, especially Asians with HBsAg <100 IU/mL and white people with HBsAg <1000 IU/mL (82). Stopping NAs treatment had no significant effects on NK cells phenotype. On the contrary, the cytotoxic reaction of NK cells increased after NAs cessation, especially in patients with HBsAg clearance. The enhancement of this cytotoxic reaction was timely related to the increase of ALT in patients, which may indicate the role played by NK cells in the clearance of HBV (83). For patients with functional cure after withdrawal of NAs, increased frequency of functional HBV specific CD8^+^T cells at baseline was related to the continued viral control after withdrawal of NAs. After the end of treatment, these HBV specific T cell responses persisted , but did not continue to increase. HBV specific CD4^+^T cell reaction has a similar trend, but there is no statistical significance (84).

### CHB functional cure and cellular immunity

HBV infection would cause damage to innate and adaptive immunity in patients. Previous studies have shown that NAs and PegIFNα offered effective complementary mechanisms of T cells immune reconstitution and NK cells immunity to better achieve the goal of clinical cure (85). In a study comparing NAs and PegIFNα combination therapy with NAs monotherapy, it was found that with the extended treatment time, the number of CD4^+^ and CD8^+^ cells decreased , but no significant difference was seen between the two groups (79). This was also in line with the previous studies that long-term treatment with PegIFNα might lead to depletion of CD8^+^T cells, showing a decreased percentage and absolute values. Additionally, the recovery of HBV-specific CD8^+^T cell function was limited (55). Therefore, suspending PegIFNα while maintaining NAs sequential and combined therapy may better assist the reconstruction of specific immunity, which may be an effective way to achieve clinical cure (86).

Further research of CD4^+^ cell subsets found that a transient increase was seen in Treg cells in the beginning, while decreased in the late stage of treatment. No significant difference was seen in the number of CD4^+^CD25^+^ and CD4^+^CD25^low^ cells between the combination therapy group and the monotherapy group, while only CD4^+^CD25^+^CD127^low^ cells increased significantly in the combination therapy group after 24 weeks of treatment (55). Several studies have shown that NK cells could secret IFN-γ to inhibit the proliferation and differentiation of Treg cells, moreover, PegIFNα could improve the inhibitory effect of NK cells on Treg cells in CHB patients (67).

As for Th cells, with the increase of treatment time, Th1 cells gradually increased, while Th17 cells gradually decreased. There was no significant difference in the number of Th1, Th2, and Th17 cells between the two groups (79). The interaction between Tfh cells and B cells was critical to humoral immunity, and in a study of patients treated with PegIFNα, the proportion of Tfh cells significantly increased after drug withdrawal in those who achieved complete response (87). Using PegIFNα as a monotherapy in another study found a increasing number of CD40L-expressing Tfh cells in patients with complete response. Tfh cells contibuted to the increase of the proportion of specific B cell subsets and the induction of antibody, thereby improving HBsAg serologic conversion rates in CHB patients (88).

Several studies have confirmed that PegIFNα treatment in children with CHB could achieve a higher HBsAg seroconversion rate and better prognosis than adults. A study of pediatric patients with CHB receiving PegIFNα treatment showed that there were significantly more activated CD8^+^ cells (CD69^+^CD8^+^, TRAIL^+^CD8^+^) and activated CD4^+^ cells (TRAIL^+^CD4^+^) in those who achieved full response compared with those who did not. The HBV-specific T cell responses of CD4^+^ and CD8^+^ continued to increase with the advancement of treatment (89). All of the above elucidated the importance of PegIFNα in promoting the activation of immune response and its close association with HBV-specific T cells, revealing part of the mechanism of inducing HBsAg serological transformation.

### CHB functional cure and humoral immune response

Targeting HBV-specific B cells as well as inducing anti-HBs antibody responses are considered as important strategies for the rational and effective CHB cure (90). Several research groups have tried different methods targeting vaccinated and CHB patients to detect the responses of HBsAg-specific memory B cell . Through the cultivation of enriched CD19^+^ cells and the stimulation of CD40-CD40L for 5 days, an HBs-ELISPOT assay was used for the identification anti-HBs secreting B cells (91). The other group used HBsAg-binding microspheres in individuals with enriched CD19+cells to directly detect HBsAg-specific memory B cells through FACS *in vitro in vitro* and showed a significant low HBsAg-specific memory B cells frequency (92). Both of these methods could effectively detect HBsAg-specific B cells in individuals inoculated with HBsAg, however, it was not true in CHB patients. Two recent studies analyzed HBsAg specific B cells in patients with the infection of HBV and used fluorochrome-labeled recombinant HBsAg as “bait” (43, 93). These two groups obtained similar results that a similarity of the frequencies of HBsAg-specific B cells can be found in acute, chronic as well as recovery phase of HBV infection, which had no linkage with HBsAg, HBV DNA and ALT levels. A longitudinal analysis showed that Tfh cells, particularly which expressed CD40L could stimulate the differentiation of B cell and improve the rate of HBsAg seroconversion in CHB patients who receiving PegIFNα monotherapy (88).

Clinical experience indicated that patients with low HBsAg levels who discontinued long-term NAs therapy (94), or shifting to PegIFNα therapy (95) can easily reach HBsAg loss. Therefore, therapeutic effect of B cells targeted vaccination could be optimized through selecting reasonably upon patients with low HBsAg levels who have been treated with NAs for a long time. Actually, a recent study enrolled 20 patients with negative HBeAg as well as HBsAg < 1000 IU/ml and found that HBsAg decreased significantly in 14 patients, and 2 patients had a HBsAg loss after inoculated conventional HBsAg-based vaccination (96). Another research showed that for HBeAg-negative patients, switching from long-term ETV therapy to adding HBsAg-based vaccination, IFNα-2b and IL-2 as a combination, there was a higher rate of HBsAg loss (9.38%) compared with patients using IFNα-2b alone (3.03%) or continuing entecavir (3.70%) treatment . Moreover, the loss rate of HBsAg was 27.3% in the combination treatment group for patients whose HBsAg titers at baseline level was in the range of 100 to 1500 IU/mL (97). These studies showed that routine HBsAg therapeutic vaccination may increase the loss of HBsAg in CHB patients treated with NAs for a long time, especially whose baseline HBsAg level were low. A recent preliminary immunization study of Sci-BVacTM selecting NAs-treated CHB patients with low HBsAg levels showed that after vaccination, three patients cleared HBsAg and developed anti-HBs . Although the study involved only a small number of patients, the result was inspiring and suggested the latent utility of this method (98).

## CHB functional cure and novel immunomodulatory agents

### CHB functional cure and host innate immune activators

PRRs agonists to activate the host’s natural immunity lead to the production/expression of proinflammatory cytokines and ISGs, which constitute an antiviral state (99). The effects of TLR-8 agonist GS-9688 on immune cell subsets were studied *in vitro* using PBMC from CHB patients and healthy participants *in vitro* and the results showed that GS-9688 could increase the frequency of CD4^+^ Tfh cells, activated NK cells, mucosal associated variant T cells and HBV specific CD8^+^T cells, as well as reduce t hat of CD4^+^ regulatory T cells and MDSCs (65). SB9200 is an activator of NOD2 and RIG-I, which can induce the activation of IFN-α/β and ISG in the blood or liver of WHV infected marmots and reduce the serum WHV DNA and WHBsAg levels. The study *in vitro* showed that activating the Recombinant Retinoic Acid Inducible Gene I Protein (RIG-I) pathway and the interaction regulatory factor (IRF) 3 induced the innate immune response which could inhibit or even eliminate cccDNA (100). Interferon gene stimulator (STING) is a major regulator of DNA mediated activation of innate immunity and a latent therapeutic target for the infection of virus. In the chronic HBV mouse model, activation of STING signal pathway can inhibit HBV replication by inhibiting the epigenetics of cccDNA, as well as attenuate HBV induced liver fibrosis by inhibiting macrophage inflammatory bodies (101). APOBEC-3 enzyme may mediate lymphotoxin and type I interferon antiviral activity through cytidine deamination, resulting in the degradation of cccDNA and participating in the host’s innate immunity to HBV (102). The α-kinase 1 (ALPK1) can activate NF- κB pathway and stimulate PRRs of innate immunity. Using mouse and primary human HBV hepatocyte models, a preclinical study investigated the anti- HBV effect of ALPK1 agonist DF-006. The results indicated that DF-006, mainly located in the liver, could activate the innate immunity in the liver and play an anti-HBV role (103).

**Figure 1:**
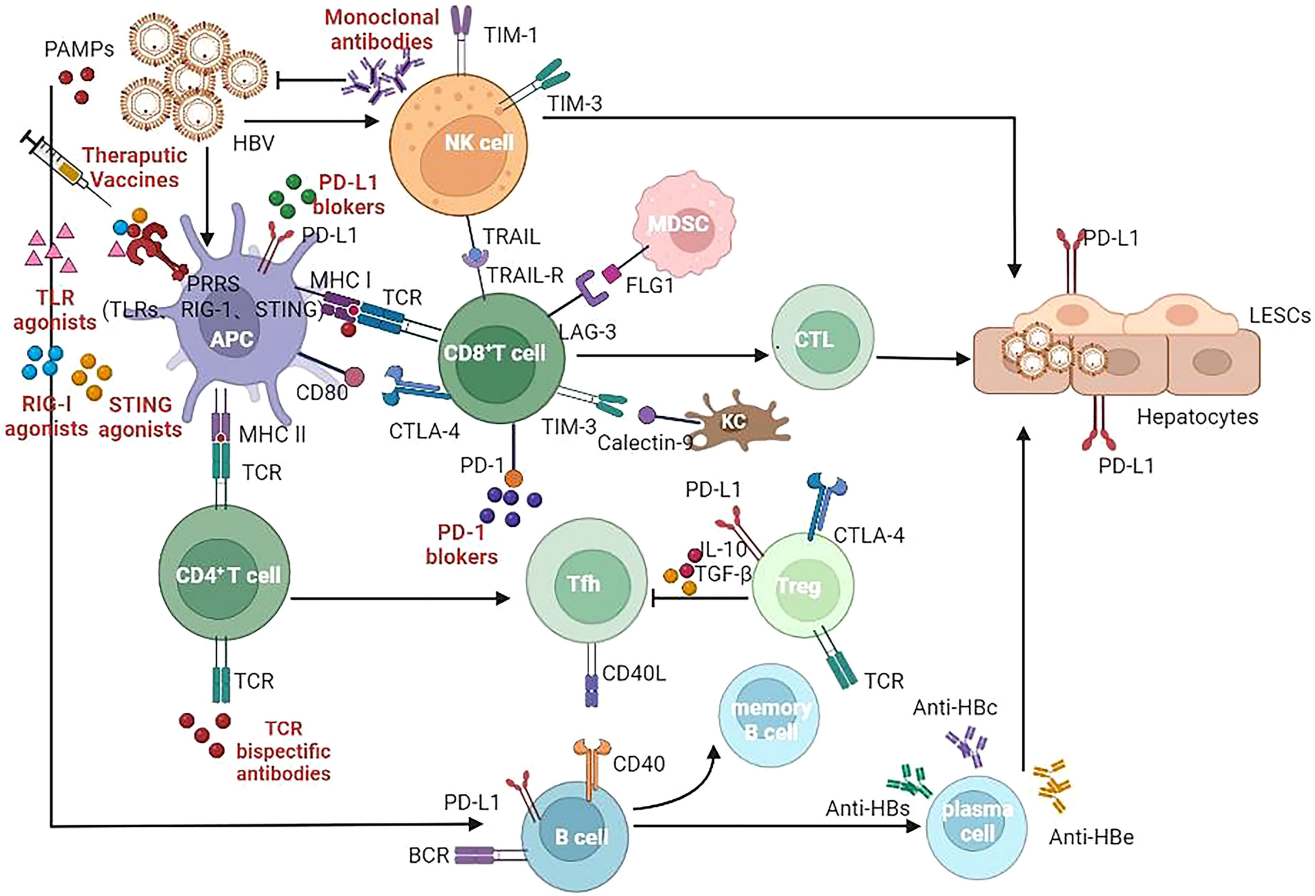
Target of immunomodulatory drugs in development for CHB infection. APC, Antigen presenting cells; NK, Natural killer (cells); MDSC, myeloid derived suppressor cells; CTL, cytotoxic lymphocyte; Tfh, T follicular helper (cells); Treg, regulatory T cells; LSEC, liver sinusoidal endothelial cells; TLR, Toll-like receptor; RIG-I, Retinoic Acid Inducible Gene I Protein; STING, stimulator of interferon genes; TCR, T cell receptor; BCR, B cell receptor; MHC, major histocompatibility complex; CLTA-4, cytotoxic T lymphocyte-associated antigen-4; PD-1, programmed death -1; PD-L1, programmed death ligand-1; TIM-3, T-cell immunoglobulin domain and mucin domain-containing molecule-3; TRAIL, TNF-related apoptosis-inducing ligand; LAG-3, Lymphocyte-activation gene 3; FLG1, Fibrinogen Like Protein 1.

Unless the subsequent adaptive immunity plays a good role, HBV infection may not be controlled only by activating the innate immunity. Therefore, understanding the characteristics of these innate immune activators not only helps to activate innate immunity, but also helps to discover their role in connecting with adaptive immunity. The role played by the immunomodulatory drugs in development of CHB infection is summarized in **Figure 1** and **Table 1** (104–129).

**Table 1:** Summary of immunomodulatory drugs in development for CHB infection.

Drug name	Developer, country	Development phase	Main research results	Reference
**Therapeutic vaccine:Induce HBV-specific B- and/or T-cell responses or generate new B/T cells**				
CVI-HBV-002	CHA Vaccine Institute, Korea	Phase IIb	—	—
GS-4774	Gobelmmune with Gilead, USA	Phase II	(Phase II) Safe and well tolerated, no significant HBsAg decreases but increased production of IFN, TNF and IL2 by CD8^+^ T cells	(104, 105)
HepTcell	Altimmune, USA	Phase II	(Phase Ib) Safe and well tolerated	(106)
VVX001	Viravaxx, Austria	Phase II	(Phase II) Safe and well tolerated, induced preS-specific IgG responses in all patients but declined during follow-up, 87% of vaccinated patients did not repeat NAs after discontinuation until end of 8-month follow-up	(107)
VBI-2601 (BRII-179)	VBI Vaccines, USA with Brii Biosciences, China	Phase IIa/IIb	(Phase Ib/IIa) Safe and well tolerated, anti-HBs responses were induced in >30% patients, moderate anti-Pre-S1 or anti-Pre-S2 antibody responses were only observed in combination with IFN-α therapy, no notable HBsAg reduction was observed	(108)
AIC649	AiCuris, Germany	Phase II	(Phase I) Safe and well tolerated, increased IL-1β, IL-6, IL-8 and IFN-γ levels, decreased IL-10 levels, the HBV DNA and HBsAg levels were essentially unchanged in most patients	(109)
GSK3528869A	GSK Biologicals, UK	Phase II	—	—
GSK4388067A	GSK Biologicals, UK	Phase II	—	—
HB-110	Ichor Medical Systems with Genexine, USA	Phase II	(Preclinical and Phase I) Safe and tolerable, induced HBV-specific T-cell responses in a portion of patients, exhibited positive effects on ALT normalization and maintenance of HBeAg seroconversion	(110)
VTP-300	Vaccitech, USA	Phase Ib/IIa	(Phase Ib/IIa) Monotherapy: induced HBV specific T cell responses and declined HBsAg only in three patients with low initial HBsAg (-0.7, -0.7 and -1.4 log_10_ respectively); Combined with low-dose nivolumab at the boosting time point: induced HBV specific T cell responses and declined HBsAg in most patients (>1 log_10_ at month 6)	(111)
VRON-0200	Viron Therapeutics, USA	Phase Ib	(Preclinical) Mouse model: elicited potent and broad CD8^+^ T cell responses, decreased HBV DNA by 2-3 log_10_, decreased HBsAg slightly (<1 log_10_)	(112)
JNJ 64300545	Janssen, Ireland with Ichor Medical Systems, USA	Phase I	—	—
JNJ 64300535	Janssen, Ireland	Phase I	(Phase I) A ≥3-fold increase in anti-HBc and/or anti-pol responses was observed in 50% of CHB patients and 91.7% of healthy individuals; HBV-specific T-cell responses were stronger in healthy individuals than CHB patients	(113)
TVAX-008	CGE HEALTHCARE, China	Phase I	—	—
CARG-201	CaroGen Crop, USA	Phase I	—	—
Chimigen HBV	Akshaya Bio Inc., Canada	Preclinical	(Preclinical) Cell model: induced vigorous T cell proliferation and enhanced expression of IFN-γ, TNF-α, perforin and granzyme B in both CD4^+^ and CD8^+^ T cell subsets; Animal model: induced a dose-dependent S1/S2-Core specific antibody response	(114, 115)
HBV	HOOKIPA Pharma, Austria, with Gilead, USA	Preclinical	—	—
TherVacB	Helmholtz Zentrum Muenchen, Germany	Preclinical	(Preclinical) Mouse model: silencing PD-L1 using siRNA enhanced the HBV-specific CD8^+^ T cell responses of TherVacB	(116)
PRGN-2013	Precigen, USA	Preclinical	—	—
ISA104	ISA Pharma, Netherlands	Preclinical	—	—
CLB-3000	Clear B Therapeutics, USA and Australia	Preclinical	(Preclinical) Mouse model: reduced HBsAg by more than 2.5 log IU/mL, 80% achieved functional cure and induced anti-HBs responses	(117)
FNX008	FANXI Biopharma, China	Preclinical	—	—
Decoy20	Indaptus Therapeutics, USA	Preclinical	—	—
**Innate immune activator: Stimulation of innate immunity effector cells and cytokines, such as T cells, IFNα, etc.**				
GS9688 (TLR8 agonist)	Gilead Sciences, USA	Phase II	(Preclinical and Phase II) Good absorption and high first pass clearance in preclinical species; reduced viral markers in HBV-infected primary human hepatocytes; combined with tenofovir alafenamide resulted in a decrease of HBsAg ≥0.5 log_10_ IU/mL in 7.4% of patients	(118, 119)
RG7854 (TLR7 agonist)	Roche, Switzerland	Phase II	(Phase I) Safe and acceptably tolerated	(120)
TQA3334 (TLR7 agonist)	ChiaTai TianQing, China	Phase II	(Phase I) Safe and tolerated	(121)
CB06 (TLR8 agonist)	ZhiMeng Biopharma, PR China	Phase I	—	—
HRS9950 (TLR8 agonist)	Hengrui Pharmaceuticals, China	Phase I	—	—
SBT8230 (TLR8 agonist)	Silverback Therapeutics, USA	Preclinical	(Preclinical) NHP: well-tolerated and efficiently targeted to the liver; Mouse model: reduced HBsAg about 2 log and drived seroconversion, lowered HBV DNA titers 2.5 logs, and induced anti-viral antigen T and B cell responses	(122)
YS-HBV-002 (TLR3, RIG1, MDA5 agonist)	YiSheng Biopharma, China	Preclinical	—	—
**Apoptosis inducer: Induce and accelerate the process of apoptosis**				
APG-1387	Ascentage Pharma, China	Phase II	(Preclinical) Mouse model: completely cleared of HBsAg, HBeAg and HBV DNA in serum, as well as HBcAg and HBV replicative intermediates in infected livers, no relapse after stop therapy; upregulated HBV-specific CD4^+^ and CD8^+^ T cells frequency and function; upregulated immune-related genes	(123)
**Monoclonal antibody: Neutralize HBV-related antigens and inhibit HBV cell entry, thereby stimulate the immune responses**				
GC1102	GC Pharma, South Korea	Phase II	(Phase I) Safe and tolerable, multiple doses of 80,000 IU and 240,000 IU resulted in HBsAg loss in 12.5% and 22.2% of patients, and all patients had baseline HBsAg ≤1 000 IU/mL	(124)
VIR-3434	Vir Biotech, USA with Brii Biosciences, China	Phase I	(Phase I) A single dose of 6 mg, 18 mg, or 75 mg induced mean HBsAg decreases of 1.30, 1.27 and 1.96 log_10_ IU/mL, but apparent rebound in HBsAg levels at week 8 was observed in all groups	(125)
162	Yangshengtang Co., Ltd, China	Phase I	—	—
BJT-778	Blue Jay Therapeutics, USA	Preclinical	—	—
KW-027	Kawin Technology, China	Preclinical	—	—
**Immune checkpoint inhibitor: Reversal of T cell exhaustion by inhibiting PD-1 or PD-L1**				
ASC22 (Anti-PD-L1)	Ascletis Pharma, PR China	Phase IIb	(Phase IIb) Reduced HBsAg by more than 0.5 log_10_ IU/mL in 21% patients and among those patients 28.6% and 14.3% achieved HBsAg seroclearance and HBsAg seroconversion, respectively	(126)
RG6084 (Anti-PD-L1)	Roche, Switzerland	Phase II	—	—
GS4224 (Anti-PD-L1)	Gilead Sciences, USA	Phase I	—	—
JNJ 63723283 (Anti-PD-L1)	Janssen, Ireland	Phase I	—	—
AB-101 (Anti-PD-L1)	Arbutus Biopharma, USA	Preclinical	(Preclinical) MC38 tumor model: reduced tumor by activating T cells with considerable potency as anti-PD-L1 antibody; PBMCs from CHB patients: reinvigorated HBV-specific T cells	(127)
ARB-272572 (Anti-PD-L1)	Arbutus Biopharma, USA	Preclinical	–	—
ALG-093453 (Anti-PD-1/PD-L1)	Aligos Therapeutics, USA	Preclinical	(Preclinical) PBMCs from CHB patients: activated HBV-specific T cells to similar or higher extend as nivolumab and durvalumab	(128)
**Other immunological candidates: Redirect nonHBV-specific T cells or stimulate innate immunity to eliminate virus-infected cells**				
IMC-I109V	Immunocore, USA	Phase I/II	(Phase I/II) Safe, decreased HBsAg levels transiently by 11–15% and then returned to baseline within 3weeks after a single low dose	(129)
LT-V11	Lion TCR, Singapore	Preclinical	—	—
TCR bispecific antibodies	Lion TCR, Singapore	Preclinical	—	—
ALVR107	AlloVir, USA	Preclinical	—	—

CHB, chronic hepatitis B; HBV, hepatitis B virus; TLR, Toll-like receptor; RIG-I, Retinoic Acid Inducible Gene I Protein; PD-L1, programmed death ligand-1.

### CHB functional cure and cellular immune activators

Based on the immune mechanism of the infection of chronic HCV, a variety of immunomodulatory drugs can strengthen the specific cellular immune response of HBV patients by addressing immunologic targets. Immune checkpoint (PD-1/PD-L1) inhibition can attenuate negative regulation of specific or resuscitate depleted T cells (130). PD-1 blockers have been proved to restore virus-specific CD8^+^T cells, but when they are depleted, blocking PD-1/PD-L1 has limited effect in CHB patients (131, 132). Other checkpoint blockers like CTLA-4 and TIM-3 also restored virus-specific CD8^+^T cells and multiple cellular immunity in CHB patients (132, 133). Lymphocyte-activation gene 3 (LAG-3), as a crucial immunomodulatory molecule, combine with MHC-class II molecules to transmit negative regulatory signals and inhibit T cell activation. Researches have found that impaired CD8^+^T cells recover better when using anti-PD-1 and anti-LAG-3 antibodies at the same time (134).

With the development of gene editing technology, edited T cells can target virus-specific immune dominant epitopes to play a long-term role in controlling HBV infection. For example, in preclinical models, CAR T cells targeting HBsAg have shown anti-HBV ability, but further research and optimization are needed for clinical application (135).

A variety of vaccines are of significant importance in enhancing cellular immune responses in patients with HBV (136). When IL-12 was used as an adjuvant, HBsAg immunization effectively reversed the systemic tolerance to HBV protein in HBV infected mice, correlated with the enhancement of HBV-specific CD4^+^/CD8^+^ T cell responses as well as the decrease of CD4^+^Foxp3^+^Treg cells (137). HBcAg-specific cytotoxic T cells are essential in controlling HBV replication, but unfortunately, vaccines containing HBcAg core 18-27 and Th epitopes are not very effective in eliminating HBV (138). On the other hand, the HBsAg/HBcAg composite vaccine developed based on the above can induce Th1/Th2 response of HBsAg, as well as maintain a balance towards Th1 type response (139). Safe and effective treatment was demonstrated in a two-year follow-up clinical study (140).

At present, there are still a variety of new immunomodulatory drugs under research, has achieved positive results. IMC-I190V is a novel bispecific protein immunotherapy that specifically clears HBsAg expressing hepatocytes infected with HBV by T cell reorientation. LT-V11 is a T-cell receptor that enhances host immunity by recruiting T cells to clear hepatocytes containing cccDNA or integrating HBV DNA. Undisclosed (CBE) can produce nonsense mutations in HBsAg and HBeAg/HBcAg ORFs, and can also cause cccDNA enrichment regions to affect HBV replication. ALVR107 is a kind of allograft under research, which aims to target HBV infected cells, and its own or allogeneic reactivity is relatively small, and has achieved very impressive early results.

### CHB functional cure and humoral immune activators

Studies have shown that adoptive transfer of anti-HBs-positive bone marrow to CHB patients can eliminate HBV in recipients, and that transfer of anti-HBs-positive donor immune cells toward HBV-infected recipients through liver transplantation can partially control HBV infection in the transplanted liver, suggesting that immune modulation is one of the essential strategies to achieve functional cure of CHB (141). Research had indicated that the acquisition and maintenance of HBsAb could be achieved through the co- activation of B cells and lineages after vaccination (142).

The likelihood of antibody mediated immunotherapy against CHB infection increased thanks to the success of polyclonal hepatitis B immunoglobulins (HBIG) (143). A pilot study involved eight amivudine-treated CHB patients treated with monthly HBIG injections and then received hepatitis B vaccination showed that after 1 year treatment, serum HBsAg decreased significantly in half of the patients. Notably, three patients were positive for anti-HBs, thus achieving a functional cure. This suggested that the combination of antiviral therapy and antibody mediated immunotherapy can trigger the B cell response after vaccination and achieved continuous HBsAg loss and functional cure (144). Zhong et al. conducted a study including 36 healthy individuals without previous history of HBV infection and HBsAb negative were given a series of three HB vaccine doses for some time of 0, 1, and 6 months with longitudinal follow-up. The result revealed that with the progress of vaccination, the overall trend of the small S protein of hepatitis B virus (SHBs) peptide coverage increased and the individual subregion recognition rate was closely related to the anti-HBs titers (145).

## Problems and prospects

IFN-based regimens have many disadvantages: inconvenient administration, long treatment duration, many and severe adverse reactions, and low functional cure rate. The host immune system is an organic whole, and the synergistic effect of natural immunity and adoptive immunity can eliminate HBV. Unlike the immunomodulatory drugs being developed that act on one or several link points of the host immune system, IFN is greatly important in both natural and adoptive immunity. Therefore, in the foreseeable future, IFN cannot be separated from the pursuit of CHB cure (146). The improvement of IFN itself (longer half-life, higher targeting of hepatocytes, fewer and lighter adverse reactions) and the optimization of IFN-based treatment scheme (higher functional cure rate) are one of the future development directions (147).

Although the research and development of new drugs are in full swing, it is far from the clinical practice. Many drugs targeting the human immune system, including therapeutic vaccines, TLR agonists, apoptosis inducers, immune checkpoint inhibitors, are in Phase 2 of clinical trials, and some are still in the preclinical exploration stage (148), The effectiveness and safety need to be further verified.

Even if novel agents go on the market, there is still a lot of work to optimize the treatment regimens. It is generally believed that the cure of CHB must be the combination of targeted HBV and host drugs, including inhibition of HBV replication, reduction of viral antigen level, immune regulation, etc. There are many drugs that in clinical trials or have been on the market in each aspect. Immune regulation involves innate immunity, specific cellular immunity, specific humoral immunity, cytokines, etc. The successful treatment of chronic HBV infection depends on not only activation of the innate immunity, but also restoration of the specific adoptive response.

Powerful and low drug resistant drugs that inhibit HBV replication have been used for many years, and one of the first-line NAs can be selected. There are many kinds of drugs to reduce the level of viral antigen, involving all aspects of viral replication cycle. The drugs regulating immune function are more. In the future, the combination of 3-4 or more drugs is likely to be required to pursue the cure of CHB. It is hoped that in the future, as in the treatment of CHC, there will be drug combinations or compound preparations with fewer times of medication, fewer tablets, fewer adverse reactions, less treatment time and higher functional cure rate.

The indications for antiviral therapy of CHB are gradually expanding. Not limited to those patients in immune clearance period (HBeAg positive) and replication period (HBeAg negative), inactive HBsAg carriers in low replication period and chronic HBV infected persons in “gray area” are gradually included in the scope of antiviral treatment. Patients in immune tolerance period begin to enter the agenda (149). Even more and more hepatologists have put forward the idea of treatment for all CHB patients. These have brought great challenges to the research and development of antiviral drugs and the exploration of treatment schemes.

Functional cure is not equal to complete cure. There is still about 10% recurrence rate and 1-2% incidence of HCC. The detection of cccDNA and integrated HBV DNA was not standardized, and the sensitivity was low. As a “hard indicator” of functional cure, HBsAg is not an ideal one for early evaluation of clinical efficacy. The search for the biomarkers closest to complete cure including immunological markers is also needed in clinical practice (150).

## Author contributions

Study concept , design and supervision (BF), acquisition, analysis and interpretation of data, drafting of the manuscript (J-RZ), critical revision of the manuscript for important intellectual content (Z-LW). All authors have made a significant contribution to this study and have approved the final manuscript.

## Funding

The work was supported in part by a grant from the National Major Project for Infectious Diseases Prevention and Treatment (No. 2017ZX10302201-004-001, 2017ZX10203202- 003-003).

## Acknowledgments

We apologize for not including all of the publications by our colleagues.

## Conflict of interest

The authors declare that the research was conducted in the absence of any commercial or financial relationships that could be construed as a potential conflict of interest.

## Publisher’s note

All claims expressed in this article are solely those of the authors and do not necessarily represent those of their affiliated organizations, or those of the publisher, the editors and the reviewers. Any product that may be evaluated in this article, or claim that may be made by its manufacturer, is not guaranteed or endorsed by the publisher.
